# Supplementary material for: The Evolution, Expression Patterns, and Domestication Selection Analysis of the Annexin Gene Family in the Barley Pan-Genome
Source: Int J Mol Sci. 2024 Mar 30;25(7):3883. doi: 10.3390/ijms25073883 (PMC11011394; doi:10.3390/ijms25073883)
Supplement: Supplementary file 1 [file ijms-25-03883-s001.zip › supplementry/supplementry file1.pdf]

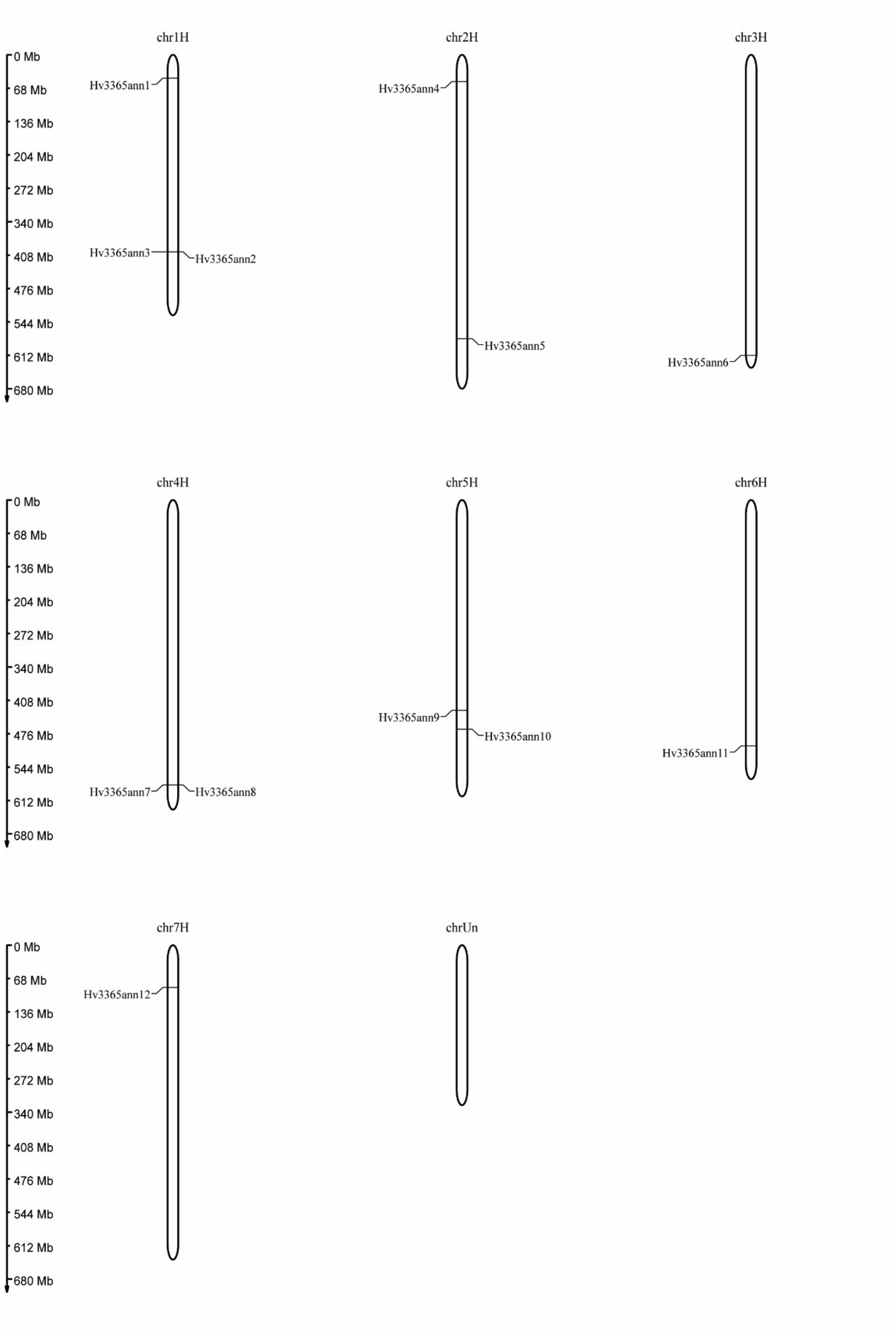

Figure S1A. physical position of Hvanns and its distribution in HOR3365.

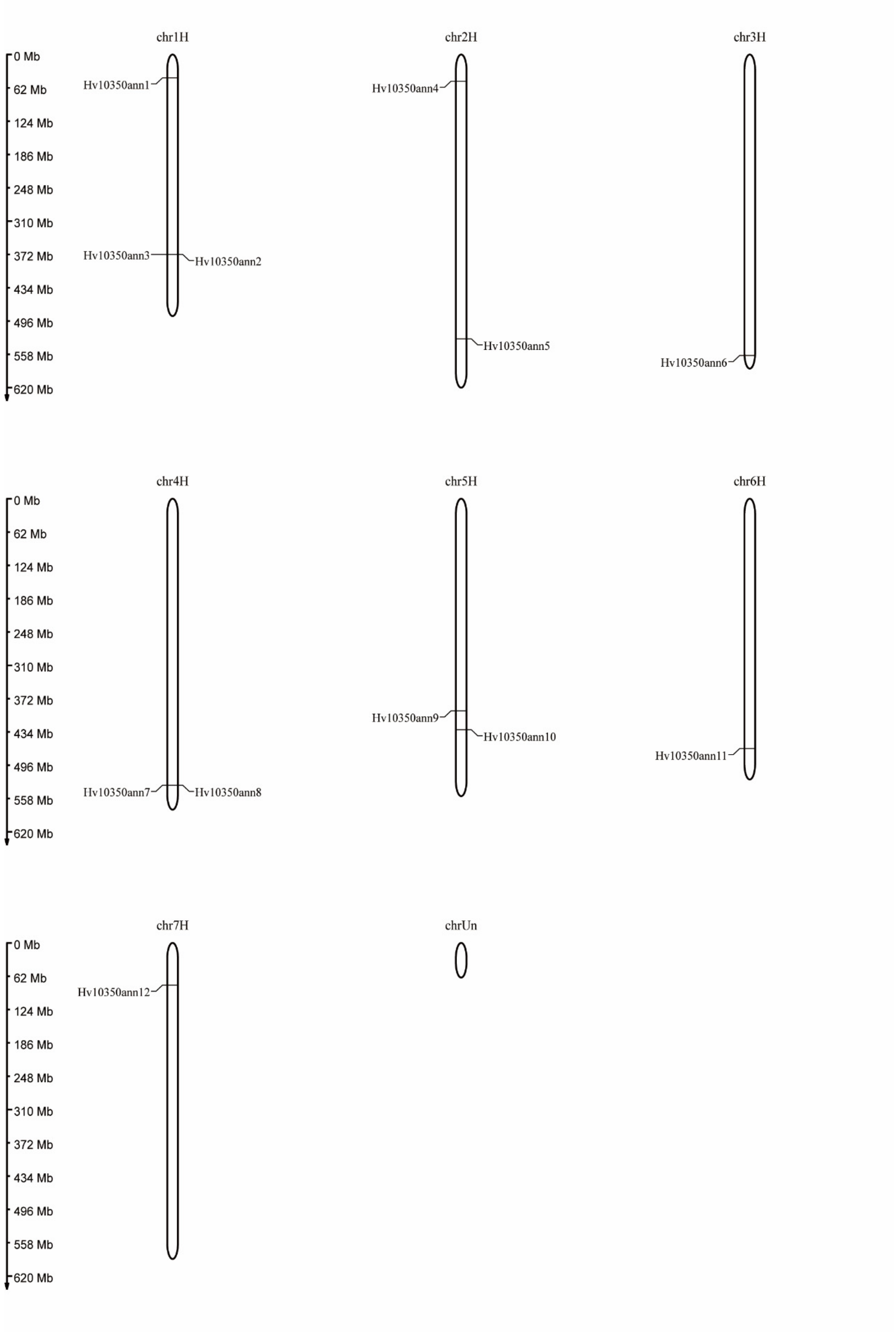

Figure S1B. physical position of Hvanns and its distribution in HOR10350.

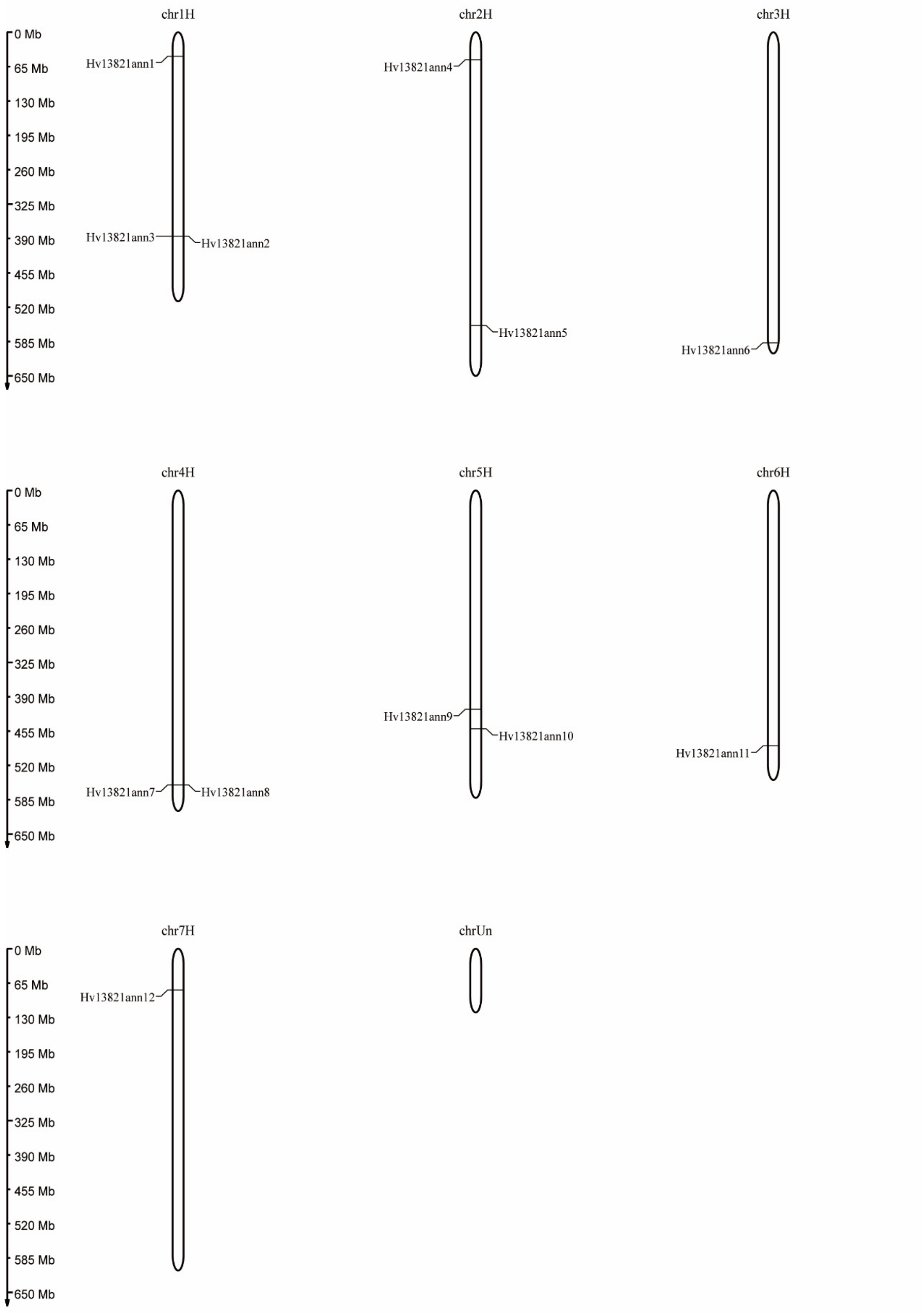

Figure S1C. physical position of Hvanns and its distribution in HOR13821.

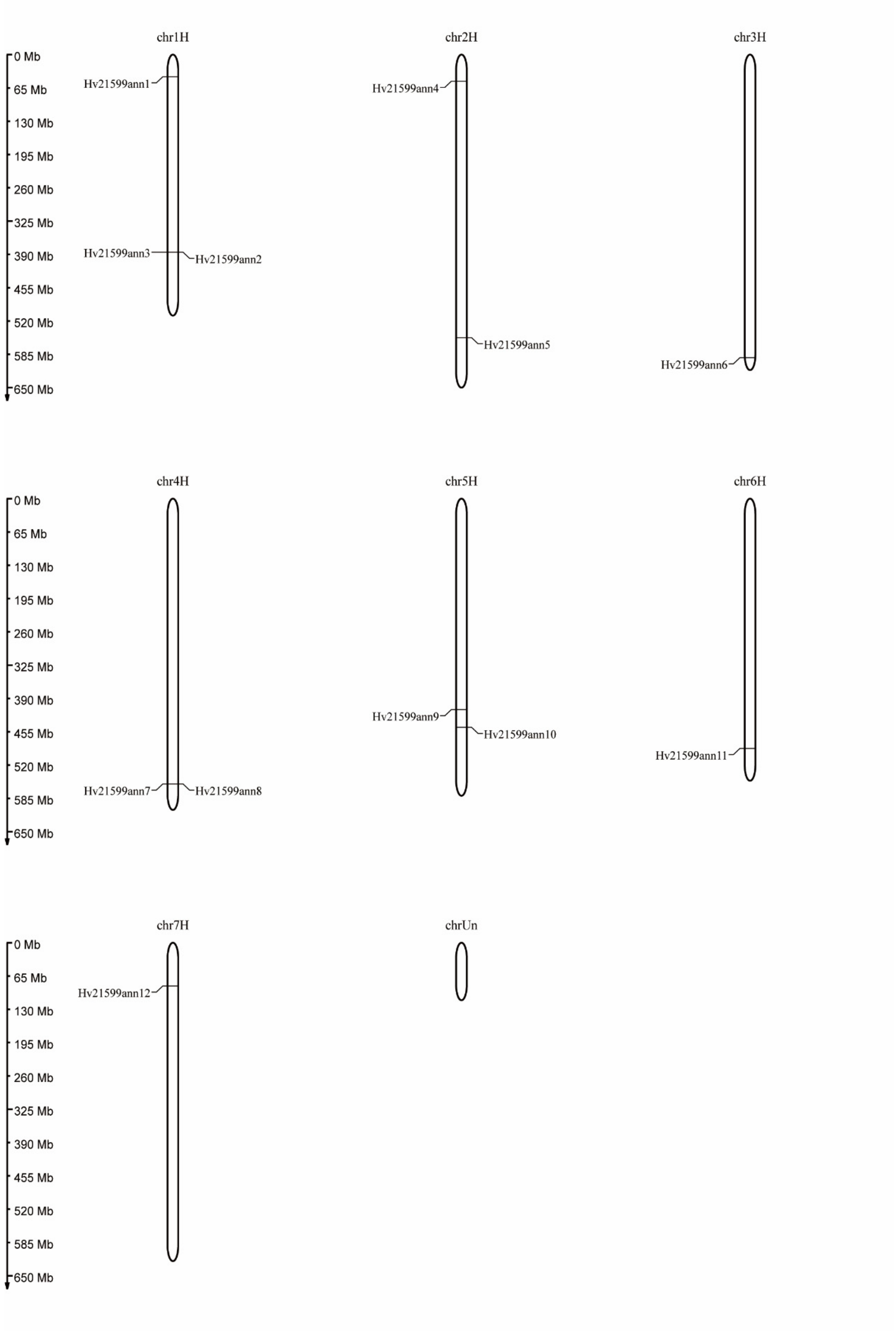

Figure S1D. physical position of Hvanns and its distribution in HOR21599.

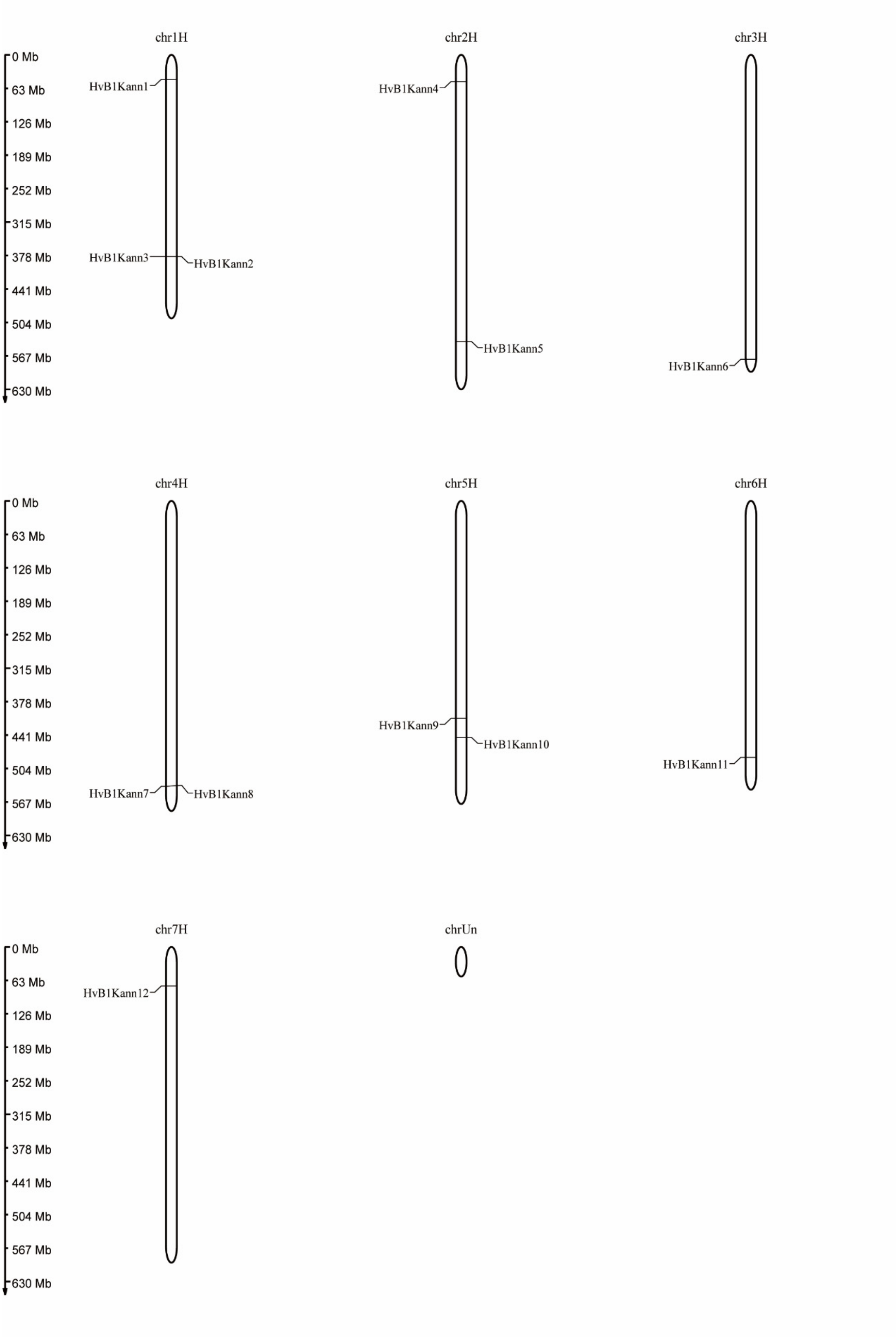

Figure S1E. physical position of Hvanns and its distribution in B1K-04-12.

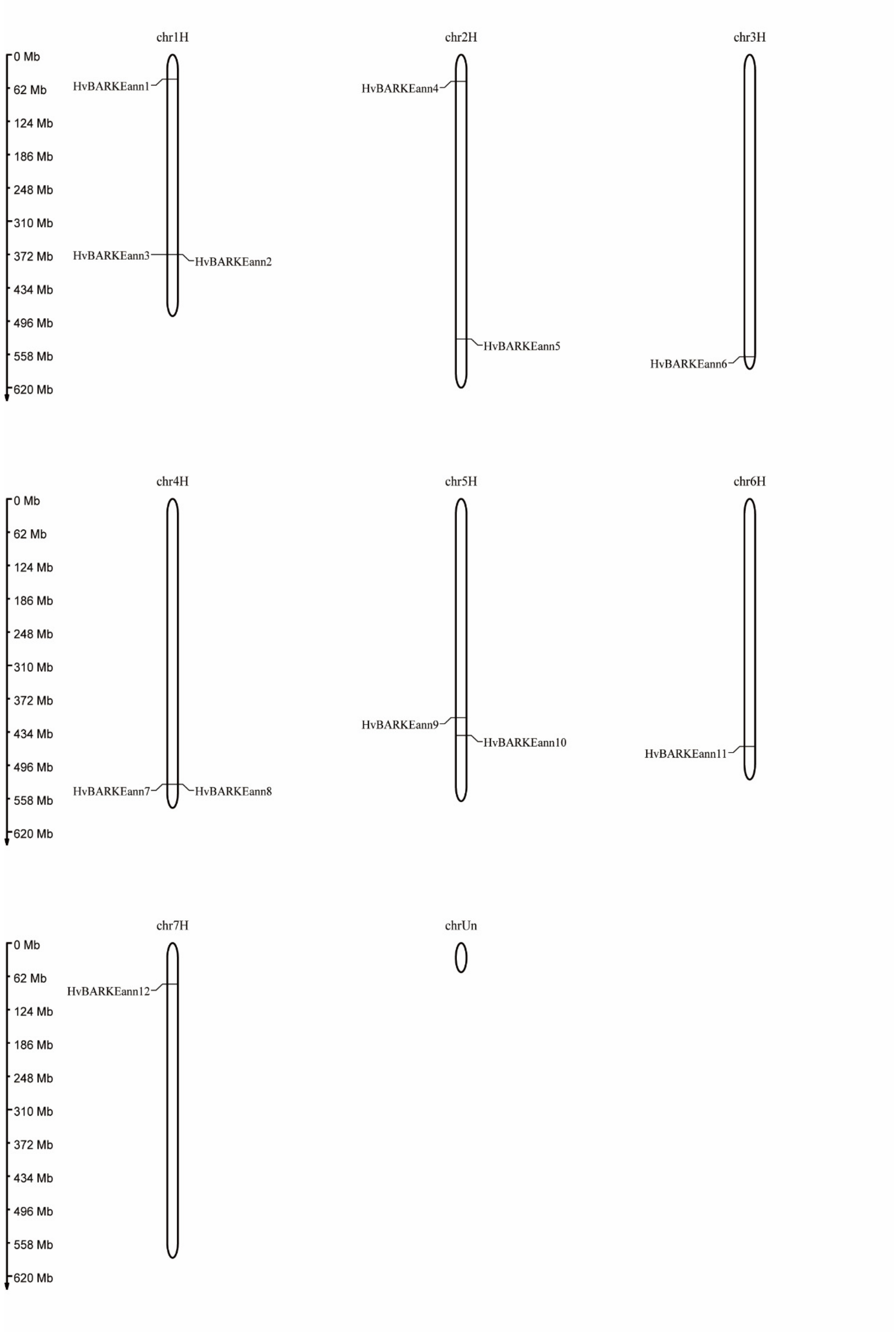

Figure S1F. physical position of Hvanns and its distribution in Barke.

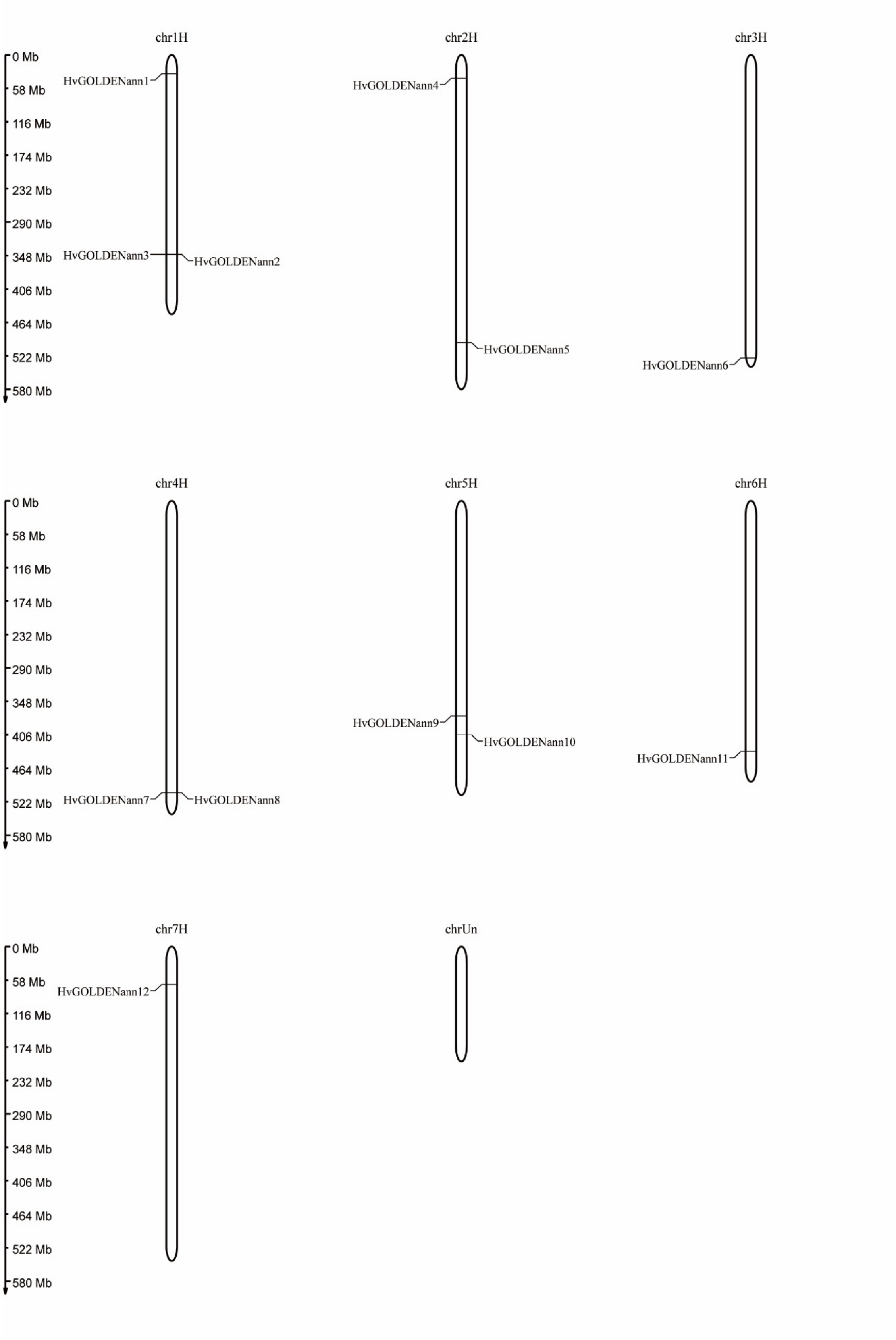

Figure S1G. physical position of Hvanns and its distribution in Golden.

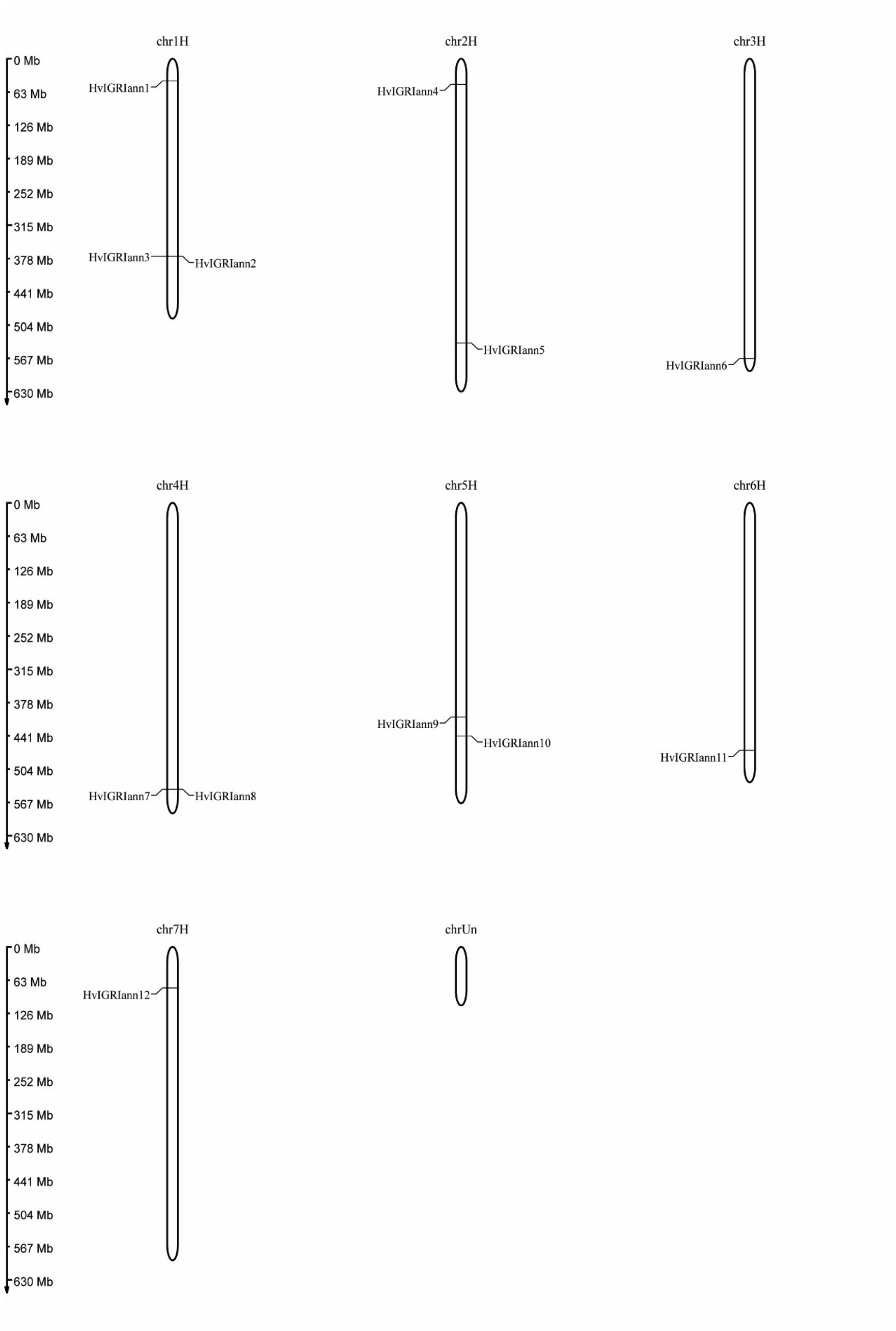

Figure S1H. physical position of Hvanns and its distribution in IGRI.

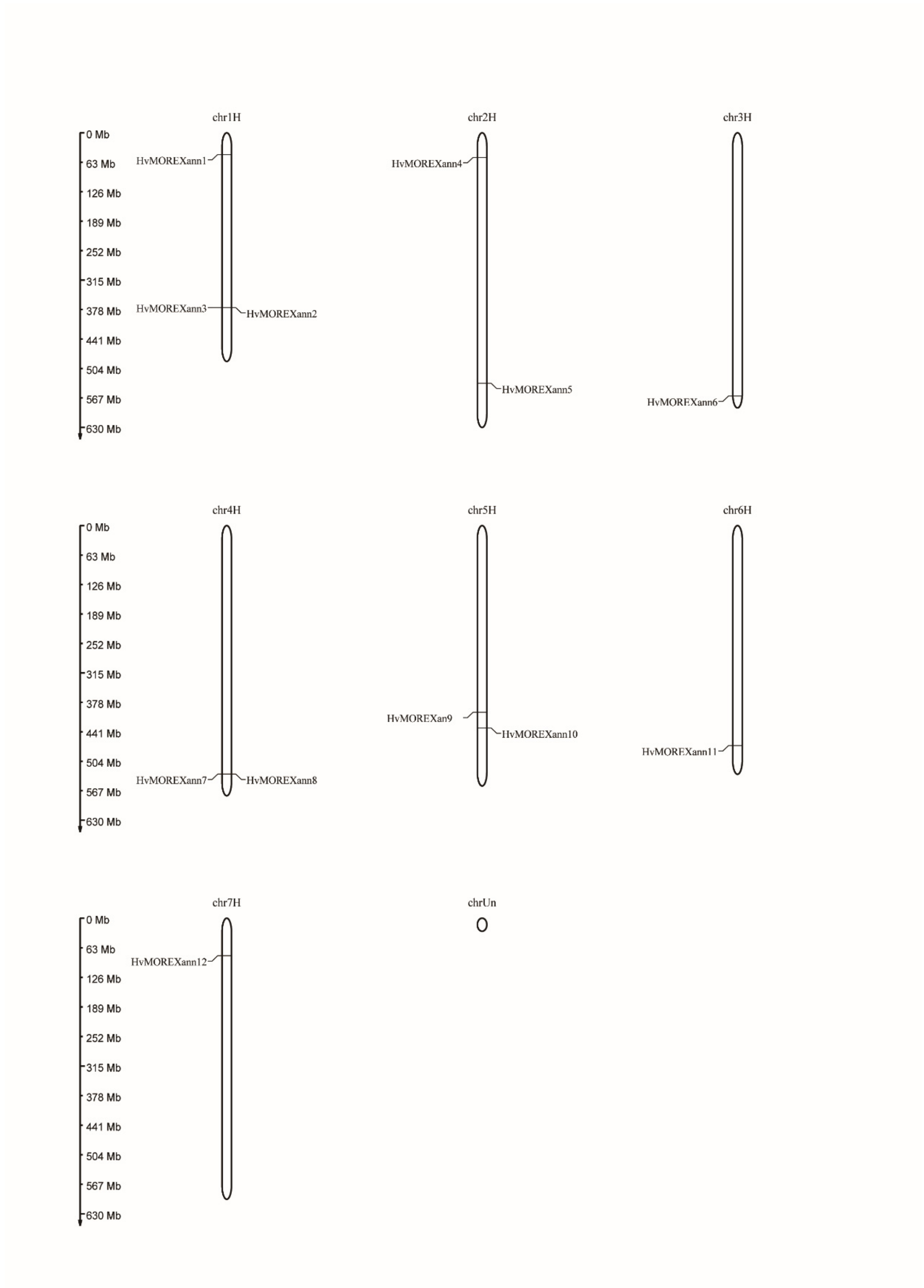

Figure S1I. physical position of Hvanns and its distribution in Morex V3.

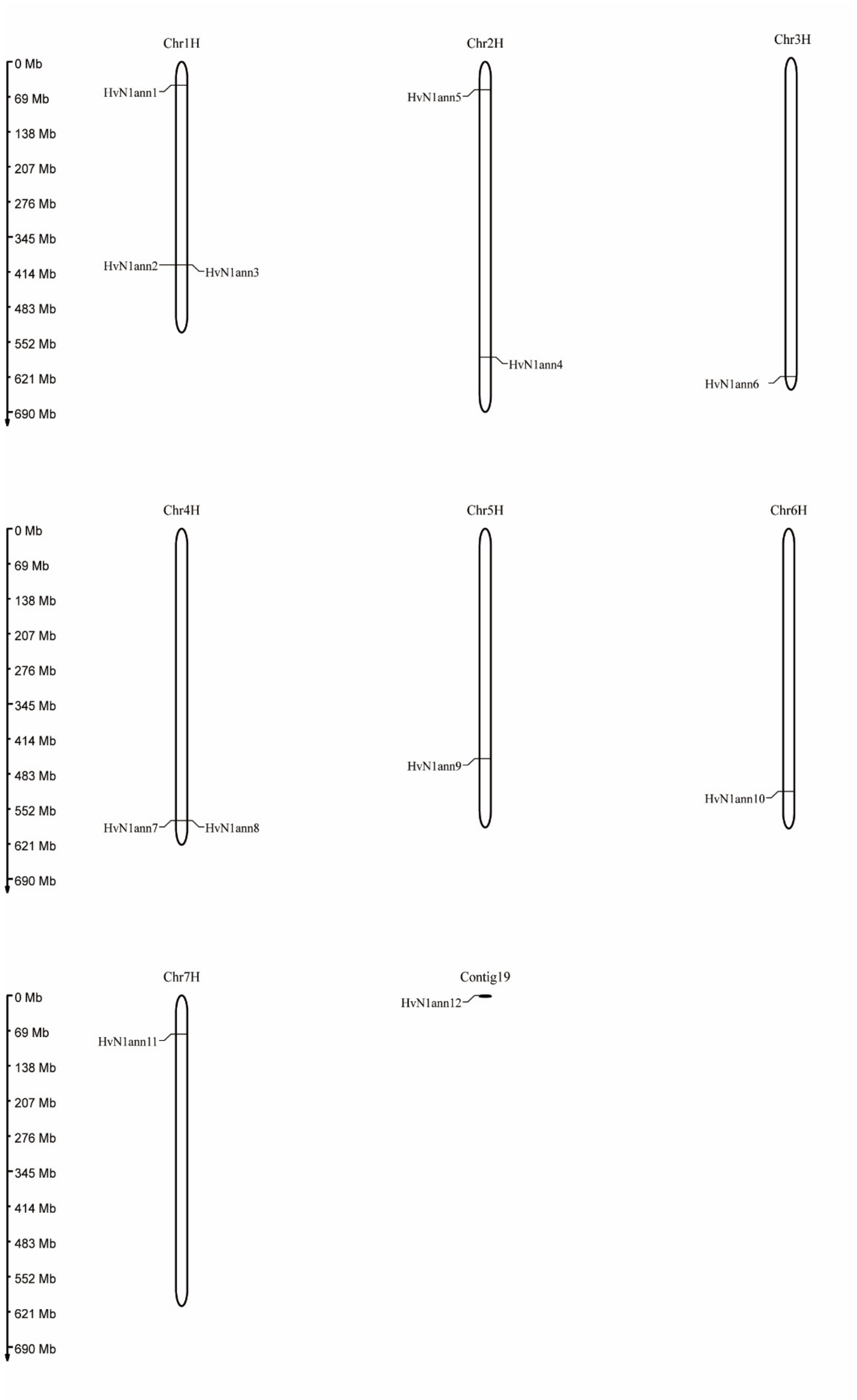

Figure S1J. physical position of Hvanns and its distribution in EC-N1.

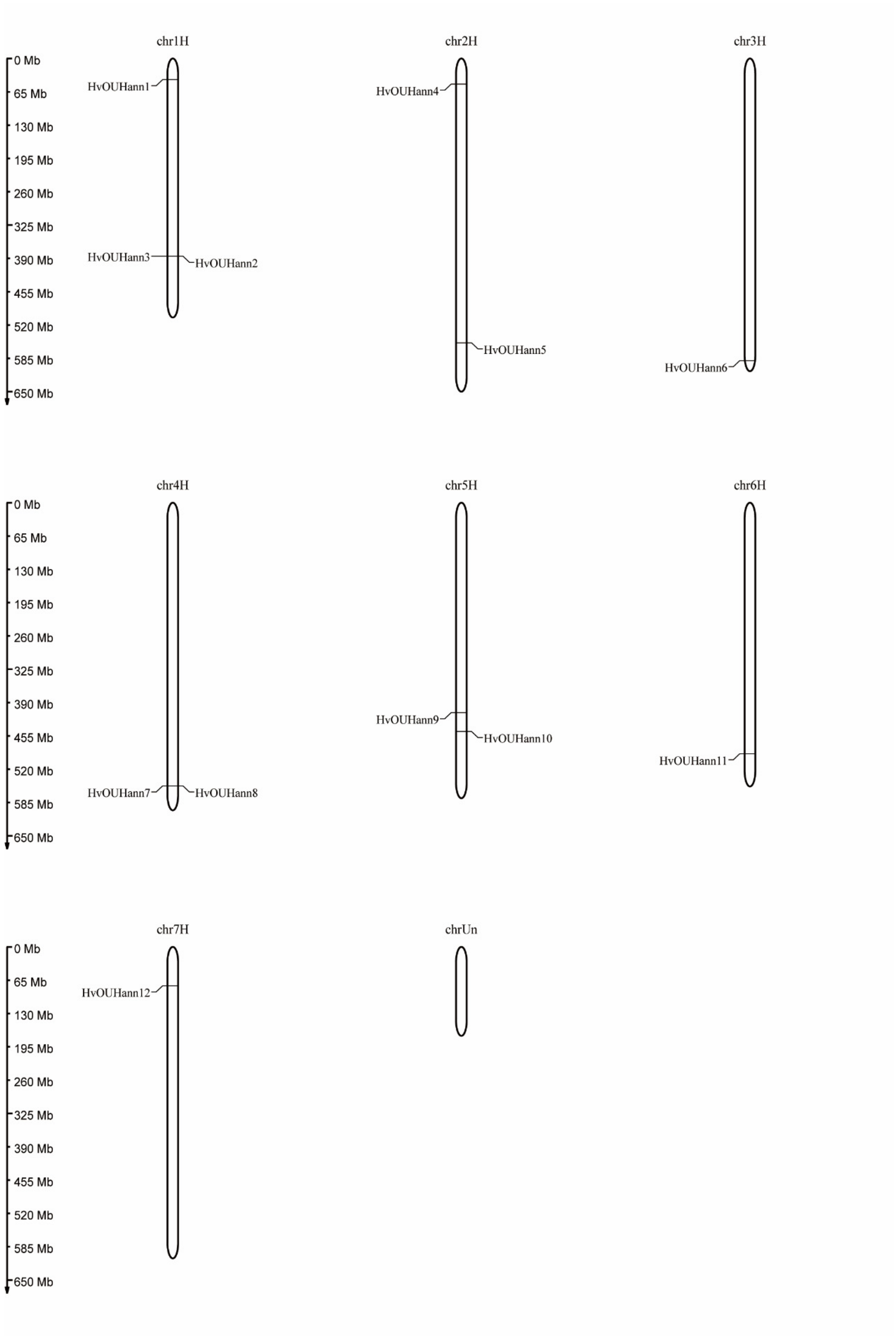

Figure S1K. physical position of Hvanns and its distribution in OUH602.

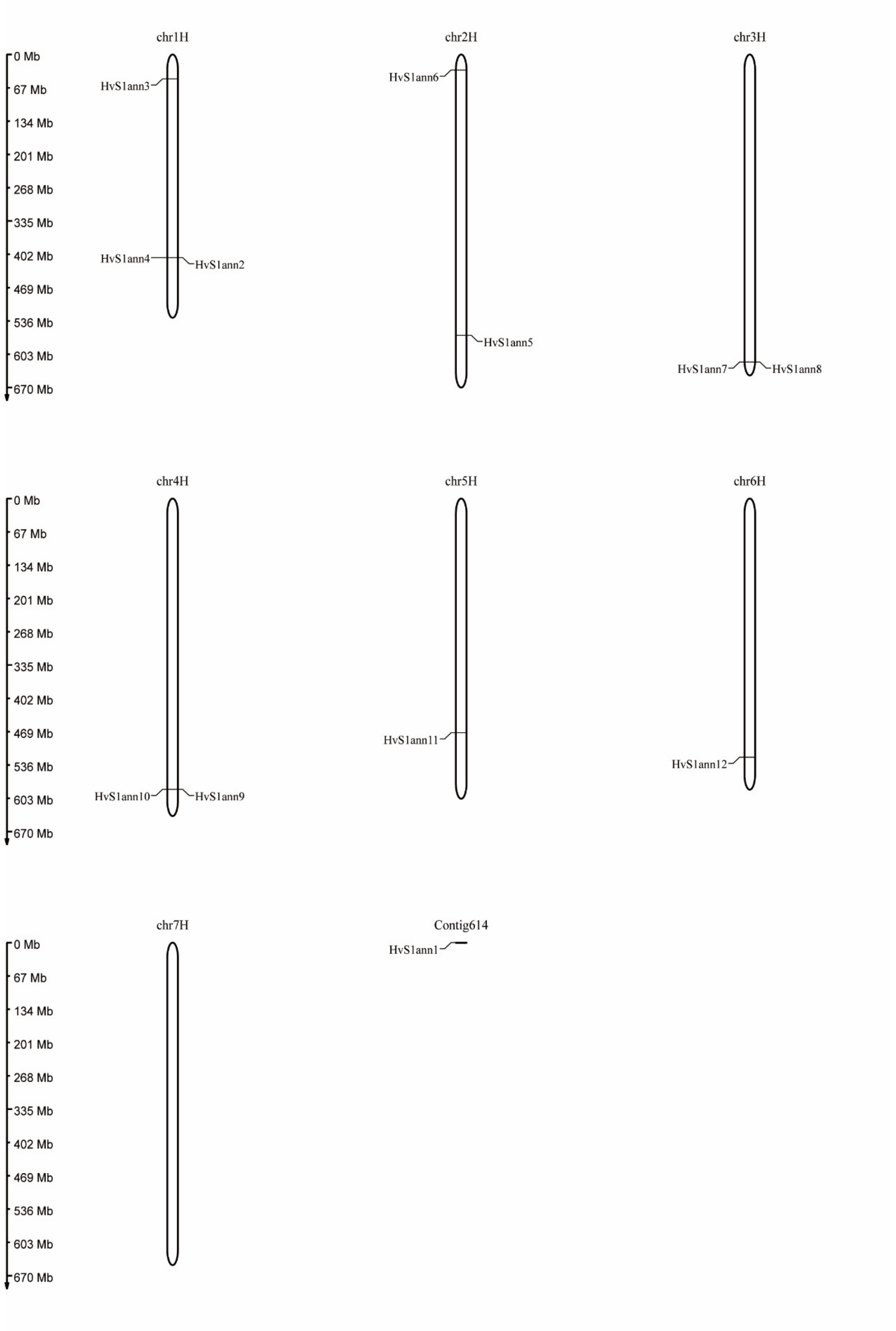

Figure S1L. physical position of Hvanns and its distribution in EC-S1.

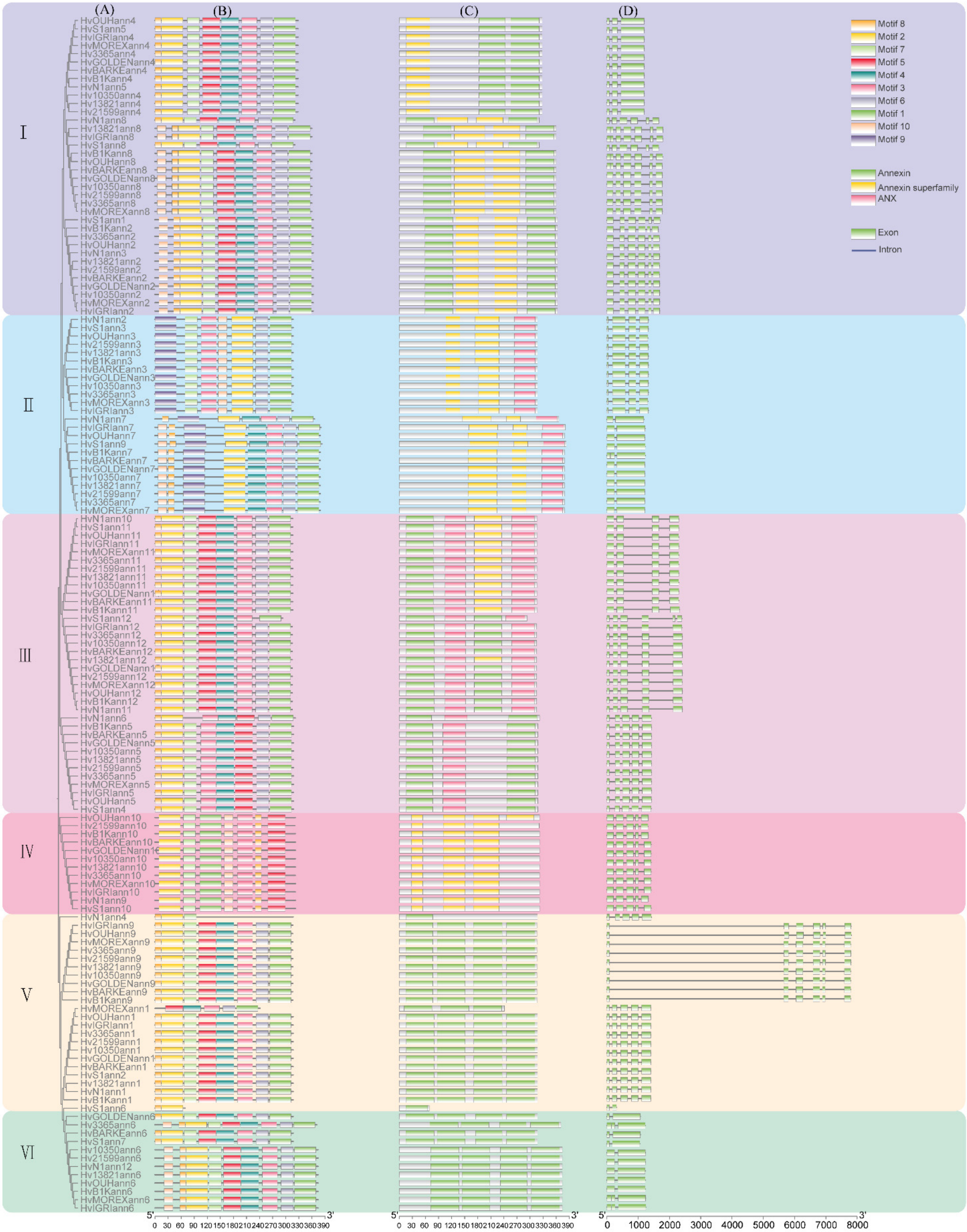

Figure S2. Motif composition, conserved domain and gene structure analysis of Hvanns. (**A**) Phylogenetic tree, (**B**) conserved motif distribution, (**C**) conserved domains,(**D**) genetic structure.

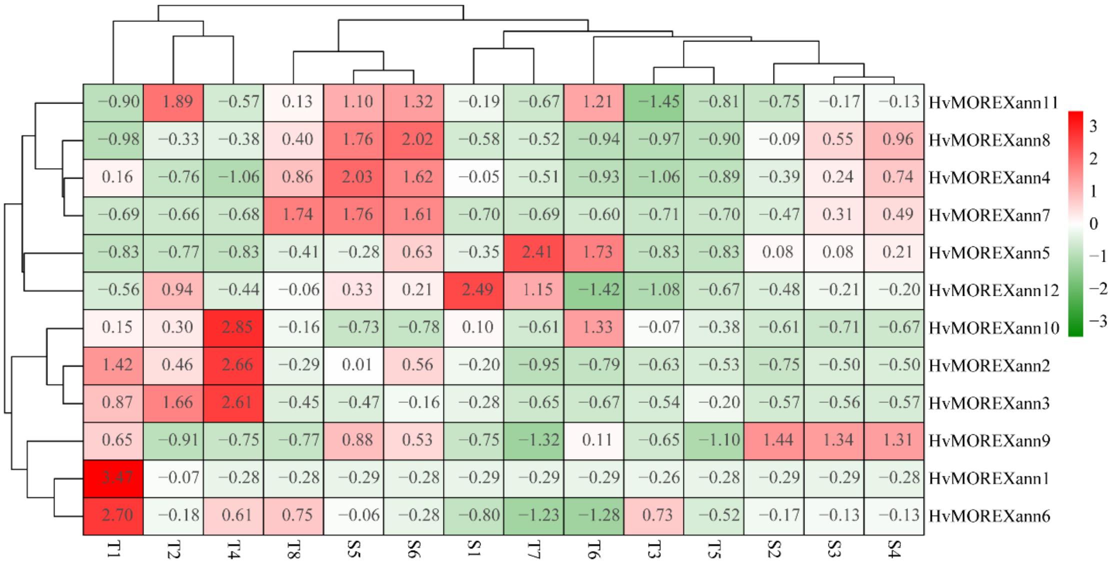

Figure S3: Transcriptome analysis of differentially expressed genes during the floret opening in barley from PRJNA752285. lodicule1(S1); lodicule2(S2); lodicule3(S3); lodicule4(S4); lodicule5(S5); lodicule6(S6); roots(T1); stems(T2); leaves(T3); rachises(T4); glumes(T5); anthers(T6); pistils(T7); lodicule(T8).

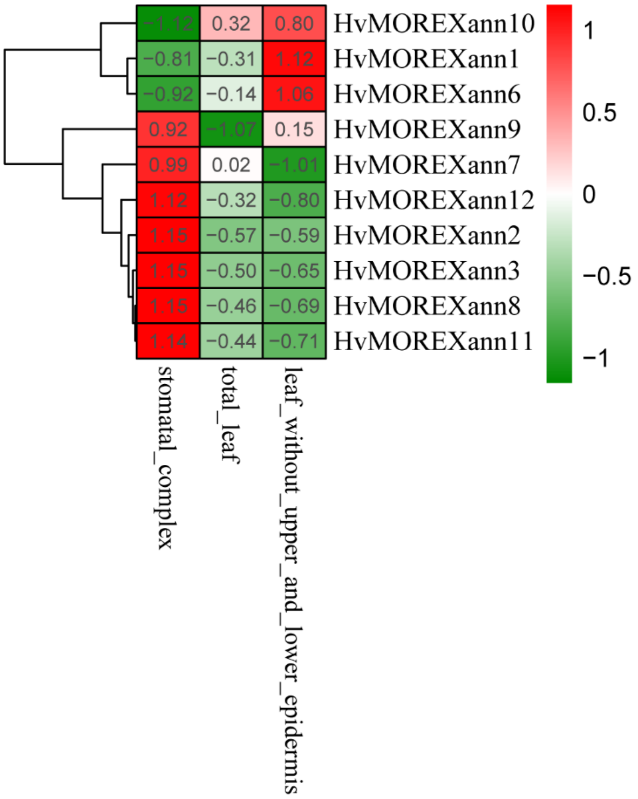

Figure S4: Transcriptome analysis of stomatal complexes and total leaves in barley from PRJEB21740.

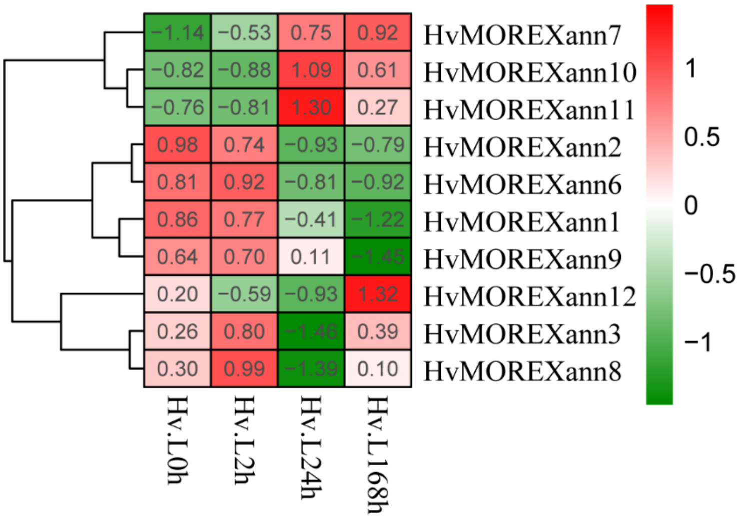

Figure S5: Transcriptome analysis of the RNA-seq datasets of monocot and eudicot plants under cold stress (4 degrees centigrade) at 0, 2, 24, and 168 hours (h) from PRJNA767196. The detailed content can be found in Table S7.

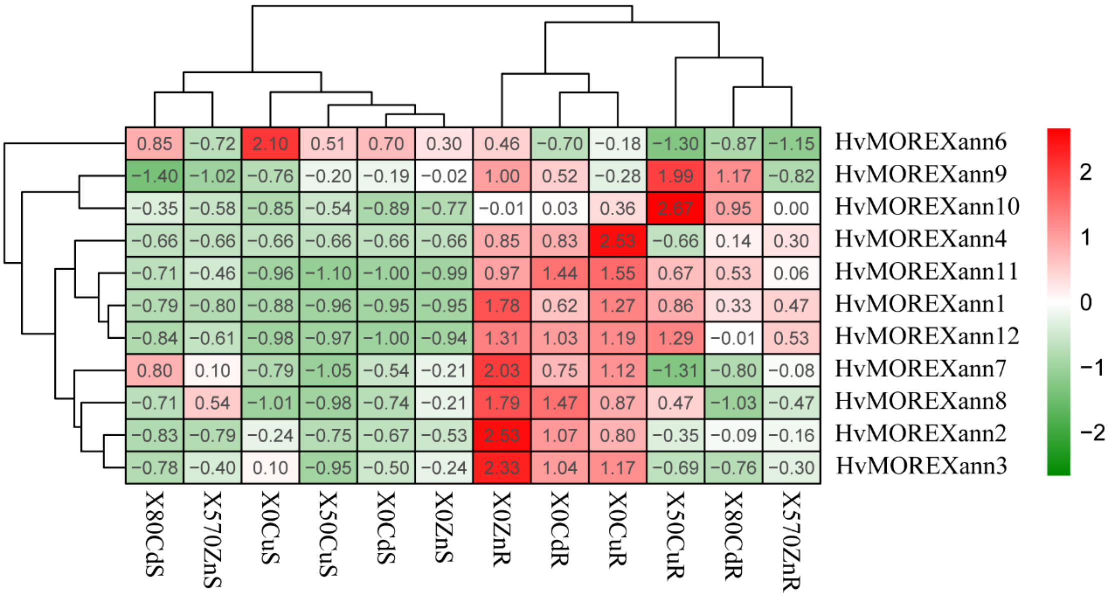

Figure S6: Transcriptome analysis of three different heavy metals Cd, Zn, and Cu from PRJNA382490. The detailed content can be found in Table S7.

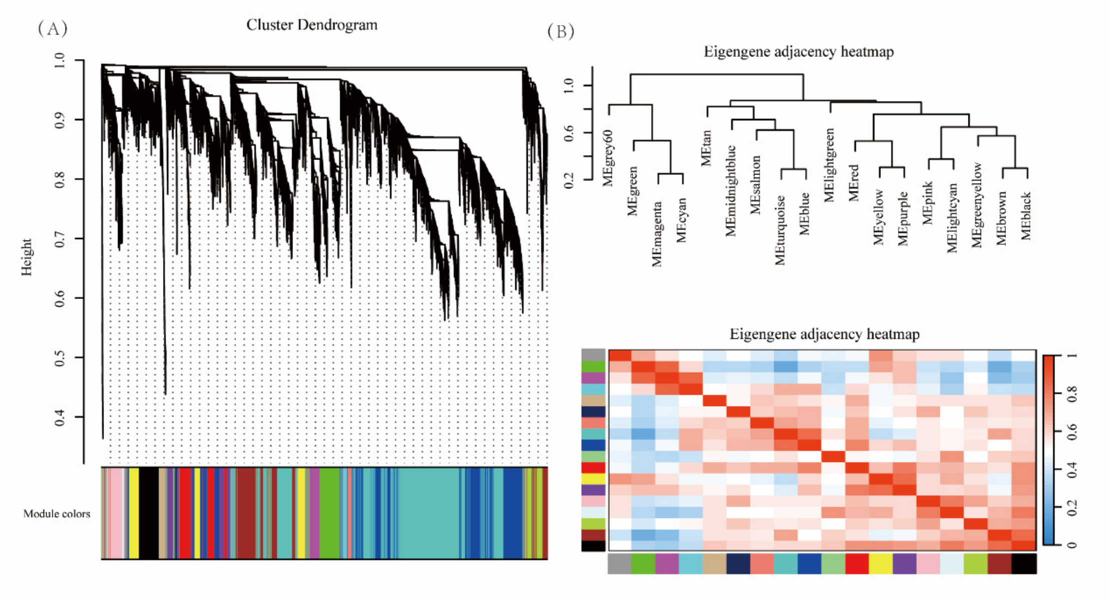

Figure S7: Cluster dendrogram(**A**) and Eigengene adjacency heatmap (**B**) for different modules from Weighted Gene Co-Expression Network Analysis (WGCNA) of Hvanns under stress treatments.
